# Cytokine Profiles of Bronchoalveolar Lavage in Patients with Interstitial Lung Diseases and Non-Allergic Asthma

**DOI:** 10.3390/ijms26146831

**Published:** 2025-07-16

**Authors:** Dana Greif Lenarčič, Urska Bidovec Stojković, Pia Kristanc, Peter Kopač, Mateja Marc Malovrh, Izidor Kern, Katarina Osolnik, Peter Korošec

**Affiliations:** 1 University Clinic of Respiratory and Allergic Diseases, 4204 Golnik, Slovenia; dana.greif@gmail.com (D.G.L.); urska.bidovec-stojkovic@klinika-golnik.si (U.B.S.); pia.kristanc@klinika-golnik.si (P.K.); peter.kopac@klinika-golnik.si (P.K.); mateja.marc@klinika-golnik.si (M.M.M.); izidor.kern@klinika-golnik.si (I.K.); katarina.osolnik@klinika-golnik.si (K.O.); 2 Medical Faculty, University of Ljubljana, 1000 Ljubljana, Slovenia; 3 Faculty of Pharmacy, University of Ljubljana, 1000 Ljubljana, Slovenia

**Keywords:** hypersensitivity pneumonitis, sarcoidosis, non-allergic asthma, amiodarone lung, EGPA, cytokines, bronchoalveolar lavage, chemokines, complement anaphylatoxins, angiogenesis-related factors

## Abstract

Diagnosing and prognosing immune-mediated airway diseases, like hypersensitivity pneumonitis (HP) and sarcoidosis, is complicated due to their overlapping symptoms and the lack of definitive biomarkers. Hence, we wanted to compare bronchoalveolar lavage (BAL) cytokine and chemokine profiles from 92 patients with different immune-mediated and inflammatory airway diseases, namely, HP, sarcoidosis, non-allergic asthma, amiodarone lung, and EGPA. We also compared pulmonary function parameters, BAL’s cellularity, and lymphocyte immunophenotypes. We found significant differences across all measured lung functions (VC, VC%, FEV1, FEV1%, and Tiff%) and in the number of macrophages, lymphocytes, neutrophils, and eosinophils. Furthermore, we showed significant differences in CD4, CD8, and CD4/8 across all included ILDs and OLDs; however, no significant differences were found in CD3, CD19, NK, or NKT. We identified nine biomarkers (IL-1β, IL-6, IL-8, IL-13, VEGF, angiogenin, C4a, RANTES, and MCP-1) that significantly differ in the BAL of patients with HP and sarcoidosis and showed that RANTES and IL-6 are associated with fibrotic outcome. We have demonstrated that interstitial and obstructive lung diseases differ in cytokine and cellular lung imprint, which may, in the future, enable the determination of the disease subtype and thus the identification of targets for the treatment of individuals or subgroups within diseases.

## 1. Introduction

Interstitial lung diseases (ILDs) and obstructive lung diseases (OLDs) are two broad categories of lung disease caused by different pathophysiological mechanisms. The term ILD encompasses acute and chronic bilateral parenchymal infiltrative lung diseases with varying degrees of tissue inflammation and fibrosis when they occur in immunocompetent hosts without infection or neoplasm [1,2]. Obstructive diseases (OLDs), including asthma, are characterized by obstructive changes caused by structural and/or functional changes in the airway wall or lumen [3].

Hypersensitivity pneumonitis (HP) results from an exaggerated immune response to environmental antigens. This leads to inflammation in the alveoli and small airways, which causes granuloma formation and may proceed to lung tissue fibrosis. In HP, after inhalation of an environmental antigen, alveolar macrophages and dendritic cells process and present the antigen to T lymphocytes. During this process, pre-inflammatory cytokines (IL-1, IL-6, TNF-α) are released and later joined by IFN-γ, IL-4, and IL-5 cytokines, which are released from T cells [4].

On the other hand, the causes of sarcoidosis remain unknown. It is believed to be a consequence of genetic predisposition, environmental triggers, and a dysregulated immune response, leading to the formation of non-caseating granulomas. The immune response is mediated by Th1 and Th17 lymphocytes, releasing cytokines like IFN-γ, IL-2, IL-6, IL-10, IL-17, and chemokines. Dysregulated regulatory T cells and impaired clearance of antigen stimuli additionally contribute to chronic inflammation and the formation of granulomas. Release of the aforementioned cytokines and chemokines can lead to fibrosis. The participation of different immune cells in developing both HP and sarcoidosis has been highlighted in several reports [4,5].

The causes of non-allergic asthma are also still mainly unclear. They vary from genetic factors to environmental pollutants, obesity, and pathogen infections. The underlying mechanisms are not entirely understood, but they involve the activation of multiple pathways in different immune cells. Among others, the mechanisms include activation of the inflammasome, release of interferons, cytokines, and chemokines from neutrophils, macrophages, mast cells, dendritic cells, and epithelial cells, and the IL-17 pathway [6]. Amiodarone lung is a consequence of taking amiodarone, a specific antiarrhythmic drug. There are two hypotheses explaining the mechanism leading to amiodarone-induced pulmonary injury. The first is that amiodarone causes direct toxic injury to lung cells, and the second is that an indirect immunologic reaction is the reason for the injury [7]. Eosinophilic granulomatosis with polyangiitis (EGPA) is characterized by asthma, eosinophilia, and granulomatous or vasculitic involvement of several organs. Its pathogenesis is driven by genetic and environmental factors (exposure to silica, organic solvents, and farming) and involves multiple cell types, the most important being eosinophils and T helper cells. Cytokine IL-5 is an effective target for the treatment of EGPA due to its importance for eosinophils’ survival [8].

Recently, immune-mediated and inflammatory airway diseases have started to be treated using biologics, targeting released cytokines, the proliferation of immune cells, and cytokine inhibitors [9,10,11,12,13,14,15,16,17,18,19]. Further data are needed to see if additional cytokine targets could be applied to subgroups of patients with these lung diseases.

Clinicians currently face difficulties in diagnosing inflammatory airway diseases, especially HP and sarcoidosis, due to their overlapping symptoms (cough, shortness of breath, chest tightness), imaging findings, and pathogenesis. HP diagnosis typically relies on clinical history, emphasizing exposure to specific antigens, characteristic symptoms, like dyspnea and cough, imaging findings showing ground-glass opacities or centrilobular nodules on high-resolution computed tomography (HRCT) scans, and laboratory tests indicating serum antibodies to offending antigens or lymphocytosis in BAL fluid [4,20].

In contrast, sarcoidosis diagnosis relies on clinical manifestations, including respiratory and systemic symptoms, such as fatigue and fever, and radiographic evidence, such as bilateral hilar lymphadenopathy visible on chest X-ray or HRCT. Confirmation through biopsy showing non-caseating granulomas remains crucial in diagnosing sarcoidosis, and BAL lymphocyte immunophenotyping might also be helpful. Sarcoidosis might also be challenging to differentiate from HP due to unknown antigenic triggers [4].

Furthermore, the diagnostic process is complicated due to the lack of definitive biomarkers. Various biomarkers have been identified, but they are not specific, and the causal relationship remains unknown, which limits their clinical use. An additional issue clinicians face is the absence of standardized diagnostic criteria and reliable diagnostic tools. All of this can lead to misdiagnosis or delayed diagnosis, which may result in disease progression and even irreversible lung damage. Therefore, finding immunological fingerprints and biomarkers appropriate for clinical use and understanding disease pathophysiology are crucial [4,21,22].

Hence, we wanted to compare the BAL cytokine and chemokine profiles of patients with inflammatory airway diseases, namely, HP, sarcoidosis, non-allergic asthma, amiodarone lung, and EGPA. We also compared the pulmonary function, BAL cellularity, and lymphocyte immunophenotypes in BAL. Herein, we show significant differences among ILDs and OLDs with regard to various cytokines (IL-1β, IL-2, IL-4, IL-5, IL-6, IL-8, IL-12p70, IL-13, TNF-α, IFN-γ), vascular remodeling-related factors (VEGF, angiogenin), complement anaphylatoxins (C3a, C4a, C5a), and chemokines (CCL5 (RANTES), CCL2 (MCP-1)), as well as pulmonary function, cellularity (macrophages, lymphocytes, neutrophils, and eosinophils), and lymphocyte immunophenotypes (CD4, CD8, and CD4/8).

## 2. Results

### 2.1. Study Participants

We prospectively sampled BAL fluid from 92 patients (Table 1) at the University Clinic of Respiratory and Allergic Diseases, Golnik, Slovenia, during a routine diagnostic bronchoscopy procedure and a routine clinical diagnostic work-up. All patients were also subjected to routine pulmonary function tests and fiberoptic bronchoscopy with biopsy in addition to BAL. The diagnoses of interstitial and obstructive lung diseases included in this study were made in agreement with the statement adopted by the joint committees of the American Thoracic Society and the European Respiratory Society. In total, 12 of the enrolled individuals were diagnosed with hypersensitivity pneumonitis (median age 68.5, 4 females), 56 were diagnosed with pulmonary sarcoidosis (median age 44.5, 27 females), 14 were diagnosed with non-allergic asthma (median age 43.5, 9 females), 5 were diagnosed with amiodarone lung (median age 74, 3 females), and 5 were diagnosed with EGPA (median age 50, 3 females). We also collected smoking information (one patient with HP, two patients with sarcoidosis, and one patient with EGPA did not want to disclose information on smoking status). All patients were monitored for at least 5 years after BAL collection for any fibrotic development.

### 2.2. Pulmonary Function and BAL Cellularity Differ Significantly Between Included ILDs and OLDs

We measured VC, VC%, FEV1, FEV1%, and Tiff% in all participants (Table 1). Data for Tiff% were not available for three patients with sarcoidosis, one patient with non-allergic asthma, and one patient with amiodarone lung. We found significant differences across all measured lung functions (VC, *p* = 0.0126; VC%, *p* = 0.0481; FEV1, *p* = 0.0003; FEV1%, *p* = 0.0273; Tiff%, *p* = 0.0262) between all included ILDs and OLDs, according to the Kruskal–Wallis statistical test. In our study, we also found statistically significant differences in BAL cellularity. We measured the number of all BAL cells, viability, and, specifically, the number of epithelial cells, macrophages, lymphocytes, neutrophils, and eosinophils. We found significant differences in the numbers of macrophages (*p* = 0.0043), lymphocytes (*p* < 0.0001), neutrophils (*p* = 0.0002), and eosinophils (*p* = 0.0052) between all included diseases. Patients with non-allergic asthma and amiodarone lung toxicity had the most macrophages and neutrophils and the least lymphocytes. Lymphocytes were prevalent in patients with HP and sarcoidosis. Patients with EGPA had the highest percentage of eosinophils.

### 2.3. Lymphocyte Immunophenotyping in BAL Showed a Significant Difference in CD4 and CD8 Lymphocytes

Lymphocyte immunotyping was routinely performed only on BAL samples where lymphocytes exceeded 15%. Therefore, BAL samples of 9 (75%) patients with HP, 35 (64.8%) patients with sarcoidosis, 2 (14.3%) patients with non-allergic asthma, 1 (20%) patient with amiodarone lung, and none with EGPA underwent lymphocyte immunophenotyping. Other samples were not immunophenotyped due to lymphocyte concentrations below 15%. We found significant differences in CD4 (*p* = 0.0459), CD8 (*p* = 0.0115), and CD4/8 (*p* = 0.0099) across all included ILDs and OLDs. CD4 was considerably higher in sarcoidosis than in other diseases; meanwhile, CD8 was the lowest in sarcoidosis. Consequently, the highest CD4/CD8 ratio was in sarcoidosis patients. There were no significant differences in CD3 (*p* = 0.1373), CD19 (*p* = 0.4972), NK (*p* = 0.3755), or NKT (*p* = 0.9581), as presented in Table 1.

### 2.4. Different Disease-Specific Cytokine Patterns in the Lungs of Patients with ILDs and OLDs

**Figure 1:**
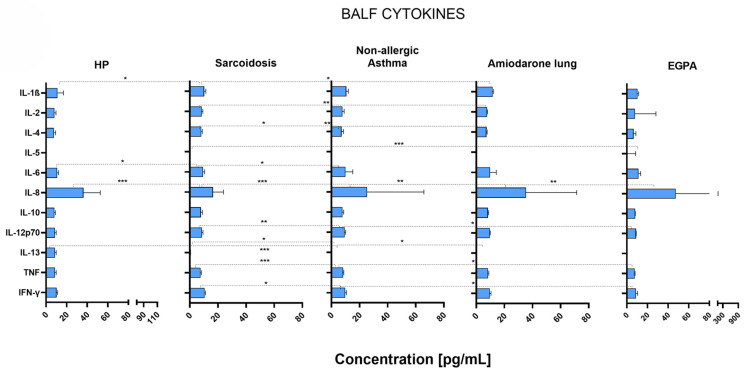
Median cytokine concentrations with IQR (defined as Q3–Q1 and represented by error bars) in the bronchoalveolar lavage fluid of patients suffering from HP (*n* = 12), sarcoidosis (*n* = 56), non-allergic asthma (*n* = 14), amiodarone lung (*n* = 5), or EGPA (*n* = 5). Correlation *p*-values were determined through the Mann–Whitney U test, and *p* < 0.05 was considered significant. Significance levels are indicated as follows: * *p* < 0.05, ** *p* < 0.01, *** *p* < 0.001.

We compared IL-1β, IL-2, IL-4, IL-5, IL-6, IL-8, IL-10, IL-12p70, IL-13, TNF-α, and IFN-γ in the BALF of patients with different ILDs and OLDs. Figure 1 summarizes the levels and differences between the included cytokines.

The levels of IL-1β were slightly lower in sarcoidosis patients than in HP (*p* = 0.04) and amiodarone lung (*p* = 0.03) patients, and the levels of IL-2 were higher in sarcoidosis patients than in amiodarone lung (*p* = 0.006) patients. The levels of IL-4 were also slightly higher in sarcoidosis patients than in non-allergic asthma (*p* = 0.02) and amiodarone lung (*p* = 0.01) patients. IL-5 was generally present in lower concentrations than other cytokines. However, the concentration of IL-5 in EGPA patients was higher than in other ILDs or OLDs, with a significant difference in comparison to sarcoidosis (*p* = 0.0002). The levels of IL-6 were lower in sarcoidosis patients compared to HP (*p* = 0.02) and non-allergic asthma (*p* = 0.02) patients. Notably, the levels of IL-8 were significantly higher in the lungs of patients with HP (*p* = 0.0007), non-allergic asthma (*p* = 0.009), amiodarone lung (*p* = 0.006), and EGPA (*p* = 0.01) in comparison to patients with sarcoidosis. There was no significant difference in the levels of IL-10 between any of the included ILDs or OLDs. The levels of IL-12p70 were higher in non-allergic asthma patients compared to sarcoidosis (*p* = 0.006) and EGPA (*p* = 0.04) patients. Cytokine IL-13 was present in higher concentrations in the lungs of HP patients than in other ILDs or OLDs and reached significance in comparison to non-allergic asthma (*p* = 0.0007) and patients with amiodarone lung (*p* = 0.02). Furthermore, patients with non-allergic asthma also had lower IL-13 levels than patients with sarcoidosis (*p* = 0.03). The levels of TNF-α were significantly higher in non-allergic asthma patients than in sarcoidosis (*p* = 0.001) and EGPA (*p* = 0.02) patients. The levels of IFN-γ were slightly higher in sarcoidosis patients than in non-allergic asthma (*p* = 0.04) and EGPA (*p* = 0.03) patients.

### 2.5. VEGF and Angiogenin Were Inversely Increased in the Lungs of Patients with Different ILDs and OLDs

**Figure 2:**
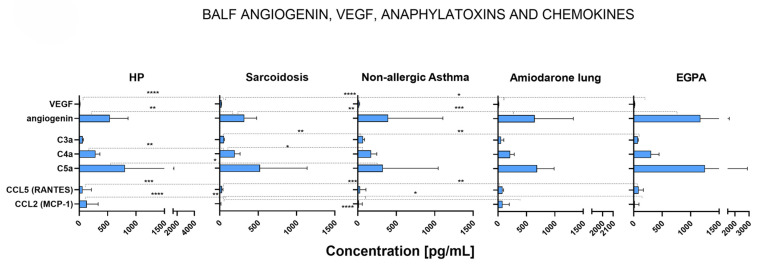
Median concentrations of angiogenin, VEGF, anaphylatoxins, and chemokines with IQR (defined as Q3–Q1 and represented by error bars) in the bronchoalveolar lavage fluid of patients suffering from HP (*n* = 12), sarcoidosis (*n* = 56), non-allergic asthma (*n* = 14), amiodarone lung (*n* = 5), or EGPA (*n* = 5). Correlation p-values were determined through the Mann–Whitney U test, and *p* < 0.05 was considered significant. Significance levels are indicated as follows: * *p* < 0.05, ** *p* < 0.01, *** *p* < 0.001, **** *p* < 0.0001.

The levels of VEGF and angiogenin were significantly different in the BALF of patients with HP, sarcoidosis, amiodarone lung, and EGPA (Figure 2).

The highest levels of VEGF were found in the lungs of sarcoidosis patients, being significantly higher than VEGF levels in HP (*p* < 0.0001), amiodarone lung (*p* < 0.0001), and EGPA patients (*p* = 0.03). On the other hand, the levels of angiogenin in the lung were higher in patients with EGPA (*p* = 0.001), amiodarone lung (*p* = 0.01), and HP (*p* = 0.007) and reached significance in comparison to patients with sarcoidosis, which showed the lowest level of angiogenin. Interestingly, there was no significant difference in vascular remodeling-related factors between non-allergic asthma patients and other ILDs or OLDs.

### 2.6. Complement Anaphylatoxins and Chemokines Were Increased or Decreased in Different ILDs and OLDs

We compared complement anaphylatoxins C3a, C4a, and C5a in the BALF of patients with different ILDs and OLDs (Figure 2). Levels of C3a were lower in sarcoidosis patients than in non-allergic asthma (*p* = 0.006) and EGPA (*p* = 0.002) patients. Levels of C4a were significantly increased in HP patients compared to sarcoidosis (*p* = 0.005) and non-allergic asthma (*p* = 0.05) patients. Additionally, levels of C4a were substantially higher in EGPA than in sarcoidosis (*p* = 0.03) patients. The levels of C5a were significantly increased in HP patients compared to non-allergic asthma (*p* = 0.04) patients.

For chemokines, we compared RANTES (CCl5) and MCP-1 (CCl2). Significant differences in chemokine levels were observed in patients with all included ILDs and OLDs (Figure 2). The levels of RANTES (CCl5) were significantly decreased in sarcoidosis patients compared to HP (*p* = 0.0003), amiodarone lung (*p* = 0.001), and EGPA (*p* = 0.005) patients. The levels of MCP-1 (CCl2) were significantly higher in HP patients than in sarcoidosis (*p* < 0.0001), non-allergic asthma (*p* = 0.008), and EGPA (*p* = 0.03) patients. The levels of MCP-1 (CCl2) were significantly higher in amiodarone lung patients than in sarcoidosis patients (*p* < 0.0001).

### 2.7. Patients with Fibrotic Outcome

HP and sarcoidosis patients were monitored for at least 5 years after bronchoscopy and phenotyping for any fibrotic outcome. The levels of cytokines, VEGF, angiogenin, complement anaphylatoxins, and chemokines were compared between patients who developed fibrotic outcomes and those who did not.

### 2.8. RANTES Is Significantly Decreased in the Lungs of HP Patients with Fibrotic Outcome

**Figure 3:**
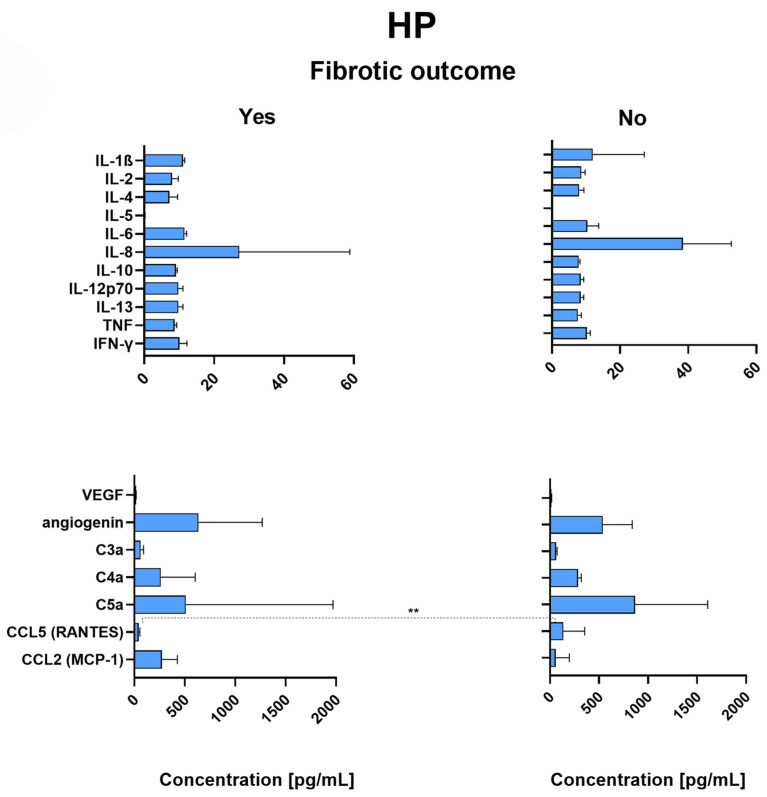
Median concentrations and IQR (defined as Q3–Q1 and represented by error bars) of all measured analytes in patients with HP either with (*n* = 4) or without (*n* = 8) fibrotic outcome. Patients were monitored for at least 5 years after bronchoscopy for fibrotic outcomes. Correlation p-values were determined through the Mann–Whitney U test, and *p* < 0.05 was considered significant. Significance level indicated: ** *p* < 0.01. HP—Hypersensitivity pneumonitis; IL—Interleukin; TNF—Tumor necrosis factor; IFN-γ—Interferon-gamma; VEGF—Vascular endothelial growth factor; C3a, C4a, C5a—Complement components 3a, 4a, and 5a; CCL5—C-C motif chemokine ligand 5; RANTES—Regulated upon Activation, Normal T cell Expressed and Secreted (alternative name for CCL5); CCL2—C-C motif chemokine ligand 2; MCP-1—Monocyte chemoattractant protein-1 (alternative name for CCL2); IQR—Interquartile range; Q1—First quartile; Q3—Third quartile.

Figure 3 shows that RANTES (CCl5) was present in significantly lower concentrations in the lungs of HP patients with fibrotic development than in HP patients without any fibrotic development (*p* = 0.008). There was no significant difference in the levels of any other measured markers between HP patients who developed a fibrotic outcome and those who did not. Nevertheless, there was a trend for IL-5, but the differences did not reach significance.

### 2.9. Sarcoidosis Patients with Fibrotic Outcome Have Significantly Increased Levels of IL-6

**Figure 4:**
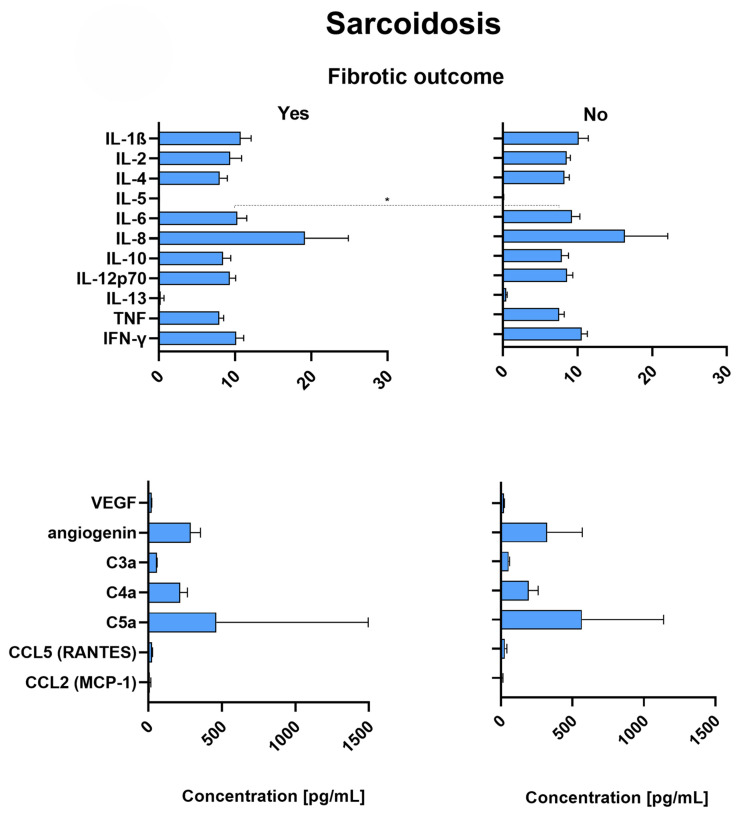
Median concentrations and IQR (defined as Q3–Q1 and represented by error bars) of all measured analytes in patients with sarcoidosis, either with (*n* = 7) or without (*n* = 49) fibrotic outcome. Patients were monitored for at least 5 years after bronchoscopy for fibrotic outcomes. Correlation *p*-values were determined through the Mann–Whitney U test, and *p* < 0.05 was considered significant. Significance level indicated: * *p* < 0.05. IL—Interleukin; TNF—Tumor necrosis factor; IFN-γ—Interferon-gamma; VEGF—Vascular endothelial growth factor; C3a, C4a, C5a—Complement components 3a, 4a, and 5a; CCL5—C-C motif chemokine ligand 5; RANTES—Regulated upon Activation, Normal T cell Expressed and Secreted (alternative name for CCL5); CCL2—C-C motif chemokine ligand 2; MCP-1—Monocyte chemoattractant protein-1 (alternative name for CCL2); IQR—Interquartile range; Q1—First quartile; Q3—Third quartile.

The levels of IL-6 were significantly increased in sarcoidosis patients with fibrotic outcome (Figure 4) compared to sarcoidosis patients without any fibrotic development (*p* = 0.03).

Besides IL-6, there were no significant differences between the cytokine levels of sarcoidosis patients with or without fibrotic development. There was also no significant difference in any angiogenesis-related factor, complement anaphylatoxin, or chemokine between sarcoidosis patients with a fibrotic outcome and those without (Figure 4).

The raw data can be downloaded from the Supplementary Materials.

## 3. Discussion

Significant differences in pulmonary function, BAL cellularity, and lymphocyte immunophenotypes among patients with HP, sarcoidosis, non-allergic asthma, amiodarone lung, and EGPA were shown in our study. Furthermore, significant differences among cytokine, vascular remodeling-related factor, complement anaphylatoxin, and chemokine profiles in the BAL of patients with different ILDs or OLDs were demonstrated.

ILD is characterized by restrictive lung function, which means that the lungs have difficulty expanding fully. This can lead to decreased lung volumes, such as vital capacity (VC) and total lung capacity (TLC), and reduced gas exchange, as indicated by the low diffusing capacity of the lungs for carbon monoxide (DLCO). In contrast, OLD is characterized by obstructed airflow due to narrowing or blockage of the airways, resulting in reduced expiratory airflow rates, such as forced expiratory volume in 1 s (FEV1) and forced vital capacity (FVC). These measures are typically reduced in diseases like chronic obstructive pulmonary disease (COPD) and asthma. Our results show that ILD is associated with restrictive lung function and impaired gas exchange, while OLD is associated with obstructive lung function and airway obstruction.

Significant differences among HP, sarcoidosis, non-allergic asthma, amiodarone lung, and EGPA patients in the quantities of macrophages, lymphocytes, neutrophils, and eosinophils were shown. The number of macrophages was the highest in amiodarone lung and non-allergic asthma and the lowest in HP. Increased numbers of lymphocytes were present in the BAL of patients with HP and sarcoidosis; meanwhile, the number of eosinophils was the lowest in these two diseases. A previous study found that patients with HP have increased lymphocyte numbers at 35.25% compared to other ILDs [23]. Neutrophil concentrations were the highest in amiodarone lung and HP and the lowest in sarcoidosis and EGPA. In ILDs, there may be increased numbers of inflammatory cells, such as lymphocytes and eosinophils [24], reflecting the underlying inflammation and immune response in the lungs. In OLDs, there may be increased numbers of neutrophils, reflecting the presence of airway inflammation and infection. When comparing lymphocyte immunophenotypes between HP and sarcoidosis patients, significant differences in CD4, CD8, and CD4/8 were observed. CD4 was increased in sarcoidosis, while CD8 was increased in non-allergic asthma and amiodarone lung and decreased in sarcoidosis. There was a two-fold difference between the ratio of CD4/CD8 in sarcoidosis patients and in patients with other diseases. The ratio of CD4/CD8 being increased in sarcoidosis was previously reported [25,26,27]. A different study showed that NK and NKT cells could have potentially different patterns in ILD, especially influencing fibrotic development. However, they did not find any significant difference in the CD4/CD8 ratio [28]. CD4 and CD8 were also reported to be connected to fibrotic development [29], which was observed in HP and sarcoidosis patients.

We found significant differences in cytokine profiles of patients with HP, sarcoidosis, non-allergic asthma, amiodarone lung, and EGPA. Patients with HP had increased levels of IL-1β, IL-6, IL-8, and IL-13. They also had decreased levels of VEGF and increased levels of angiogenin, C4a, C5a, RANTES, and MCP-1. HP patients with fibrotic development during the 5-year period after initial diagnostics had significantly lower concentrations of RANTES than HP patients without any fibrotic development. Sarcoidosis patients had decreased levels of IL-1β, IL-5, IL-8, IL-12p70, TNF-α, angiogenin, C3a, C4a, RANTES, and MCP-1 and increased levels of IL-2, IL-4, IL-6, IL-13, IFN-γ, and VEGF. Decreased levels of IL-8 and increased levels of VEGF in sarcoidosis compared to HP were previously reported, thus agreeing with our findings [26]. IL-8 was found to be important in the development of ILD [30]. The levels of IL-6 were significantly higher in sarcoidosis patients with fibrotic outcome than those without, which is in line with the previous finding that IL-6 has a protective role against lung fibrosis [31]. In the BAL of patients with non-allergic asthma, we measured decreased concentrations of IL-4, IL-8, IL-13, IFN-γ, C4a, C5a, and MCP-1 and increased concentrations of IL-6, IL-12p70, TNF-α, and C3a. Decreased levels of IL-4 in asthma patients have been reported before, despite it being a regulator of IgE synthesis in vitro [32]. Patients with amiodarone lung had increased levels of IL-1β, IL-8, angiogenin, RANTES, and MCP-1 and decreased levels of IL-2, IL-4, IL-13, and VEGF. Lastly, EGPA patients had increased levels of IL-5, IL-8, angiogenin, C3a, C4a, and RANTES and decreased levels of IL-12p70, TNF-α, IFN-γ, VEGF, and MCP-1. Increased levels of IL-5 might cause enhanced eosinopoiesis, eosinophil maturation, activation, and prolonged survival, characteristics of EGPA [33]. Different profiles reflect diseases’ pathophysiological mechanisms and the involvement of distinct cells that mediate the immune response [20,34].

Based on our results, 9 possible biomarkers were identified (IL-1β, IL-6, IL-8, IL-13, VEGF, angiogenin, C4a, RANTES, and MCP-1) out of 18 tested that significantly differ in the BALF of patients with HP and sarcoidosis. These could be used in the process of diagnosing, as HP and sarcoidosis often have overlapping symptoms and are difficult to differentiate [4]. It seems that differences in the concentrations of these cytokines in bronchoalveolar lavage could also influence the response to therapy or the course of the disease [12]. The difference in cytokine profiles of HP and sarcoidosis patients could result from HP being associated with innate immunity, mediated by monocytes and alveolitis, while sarcoidosis is linked to Th1/Th17-driven granuloma formation and T cell involvement [4].

Analysis of bronchoalveolar lavage is a useful tool for the diagnosis of interstitial and obstructive lung diseases. Bronchoalveolar lavage is a procedure performed with a fiberoptic bronchoscope within the selected bronchopulmonary segment. Examination of cells and fluids from the lower respiratory tract provides valuable information about the diagnosis and gives insight into the immunological, inflammatory, and infectious processes taking place at the alveolar level [4].

We have demonstrated that interstitial and obstructive lung diseases differ in cytokine and cellular lung imprint, which could enable the determination of the disease subtype and thus the identification of targets for the treatment of individuals or subgroups within diseases. However, there are several limitations to our study. Some of the analyses were carried out in relatively small subgroups (amiodarone lung and EGPA) and may therefore not be sufficiently powered to detect differences between the groups, and we cannot exclude the possibility of a Type II error. Additional larger studies should be completed to confirm the findings and further research potential biomarkers for clinical use. Another limitation is that the capacity for lymphocyte phenotyping is constrained in BAL samples, which limits the accuracy of our analyses. However, our study still included more subjects than other BAL studies generally do. Furthermore, coupling BAL samples from multiple timepoints would have enabled us to monitor for any changes; however, that was not possible due to bronchoscopy being an invasive procedure. Finally, the lack of a control group of healthy individuals, again due to bronchoscopy being an invasive procedure, is another limitation. However, due to the main issue being overlapping symptoms and clinicians struggling to differentiate between the diseases, comparing biomarker profiles among said diseases is central for an objective and confident diagnosis.

## 4. Materials and Methods

### 4.1. Research Design and Protocol

Analysis of cellular and cytokine profiles was performed on bronchoalveolar lavage. All patients gave written consent, and the study was approved by the Medical Ethics Commission of the Republic of Slovenia (No. 0120–271/2018/4). We included patients with asthma, hypersensitivity pneumonitis, sarcoidosis, amiodarone lung, and EGPA. For all patients, we compared the results of pulmonary function (VC, VC%, FEV1, FEV1%, and Tiff%), cytology in bronchoalveolar lavage (epithelial, macrophages, lymphocytes, neutrophils, and eosinophils), cytokines, factors related to angiogenesis (VEGF and angiogenin), anaphylatoxins (C3a, C4a, and C5a) and chemokines CCL5 (RANTES), CCL2 (MCP-1) in bronchoalveolar lavage supernatant, and lymphocytes in bronchoalveolar lavage (CD3, CD4, CD8, CD4/8 index, CD19, and NK and NKT cells).

### 4.2. Methods

#### 4.2.1. BAL Procedure

Bronchoalveolar lavage was performed during bronchoscopy with a flexible instrument (Olympus, Tokyo, Japan) with 150 mL of physiological solution heated to 37 °C in one of the segmental bronchi of the middle lobe. The physiological solution was applied 7 times at 20 mL and 1 time at 10 mL and immediately aspirated into a bottle that was transported to the laboratory on ice. After filtering the bronchoalveolar lavage through sterile gauze and then a 70 µm cell filter (BD FACSCanto™ II (BD Biosciences, Franklin Lakes, NJ, USA)), we centrifuged it and resuspended the cells in the Hemacell. Cell smears and counting and cytological differentiation of cells were prepared for all bronchoalveolar lavages.

#### 4.2.2. Measurements of Cytokines (IL-1β, IL-2, IL-4, IL-5, IL-6, IL-8, IL-10, IL-12p70, IL-13, TNF-α, IFN-γ), Factors Related to Angiogenesis (VEGF, Angiogenin), Anaphylatoxins (C3a, C4a, C5a) and Chemokines CCL5 (RANTES), and CCL2 (MCP-1) in the Supernatant of Bronchoalveolar Lavage

The following reagents and equipment were used for analysis: BD Cytometric Bead Array (CBA) and FACS Canto II flow cytometer (BD, USA). In the assay, soluble analytes/sets of analytes are captured on microbeads of known fluorescence and size, allowing for detection through flow cytometry. Each capture bead has a specific fluorescence and is coated with an antibody that is specific to an individual protein (analyte). When capture beads and detection reagent are incubated with standards or unknown samples, sandwich complexes are formed. These complexes were highly sensitive and quantitatively measured with a flow cytometer. Data analysis was performed with the FCAP Array program (BD, Franklin Lakes, NJ, USA).

#### 4.2.3. Flow Cytometric Analysis of Lymphocytes in Bronchoalveolar Lavage

For the analysis, we used different mouse monoclonal antibodies that recognize antigenic determinants, CD3, CD4, CD8, CD14, CD16+56, CD19, and CD45 (BD, USA), labeled with FITC, PE, or PerCP and APC. The cells from the bronchoalveolar lavage were labeled with different combinations of four different antibodies. Lymphocytes were separated from other cells through two-color marking based on size (FCS parameter), granulation (SCC parameter), and CD45 marking. At least 2000 lymphocytes were analyzed in each test tube. Measurements and analysis were performed with a FACS Canto II flow cytometer and BD FACSDiva (version 8.0.1) (BD Biosciences, Franklin Lakes, NJ, USA).

#### 4.2.4. Statistical Analysis

In the analysis, quantitative variables are expressed as medians and the interquartile ranges.

In the study and statistical analysis of the results, we included only patients who had undergone bronchoscopy as part of the diagnosis of the underlying disease, and none of the included individuals had been on a systemic steroid administered for at least one week before bronchoscopy. We tested the assumption of equality of mean values in individual analytes in bronchoalveolar lavage between independent groups (HP, asthma, sarcoidosis, EGPA, and amiodarone lungs) using the Kruskal–Wallis test. With the Mann–Whitney U-Test, we compared the differences between all independent groups. We performed this part of the analysis with the GraphPad program.

## Figures and Tables

**Table 1:** Demographics, clinical characteristics, pulmonary function, BAL cellularity, and lymphocyte immunophenotyping of the study cohort. Lymphocyte immunophenotyping was performed on samples with lymphocyte counts exceeding 15%.

Characteristic	HP (*n* = 12)	Sarcoidosis (*n* = 56)	Non-Allergic Asthma (*n* = 14)	Amiodarone Lung (*n* = 5)	EGPA (*n* = 5)	*p* Value *
Age [years], median (range)	68.5 (49–81)	44.5 (25–79)	43.5 (19–71)	74 (65–79)	50 (21–80)	<0.0001
Sex, no. (%)						0.5666
Male	8 (66.7%)	29 (51.8%)	5 (35.7%)	2 (40.0%)	2 (40.0%)	
Female	4 (33.3%)	27 (48.2%)	9 (64.3%)	3 (60.0%)	3 (60.0%)	
Smoking						0.6500
Yes	0	5	0	0	0	
No	10	43	11	5	4	
Ex	1	6	3	0	0	
Pulmonary function, median (range)						
VC	3400 (600–5350)	4255 (1800–7000)	3085 (1640–5200)	2400 (1600–3800)	3060 (2500–4150)	0.0126
VC%	97 (57–132)	94 (63–123)	85 (53–127)	80 (26–93)	71 (67–94)	0.0481
FEV1	2010 (1100–4000)	3335 (1020–5150)	2325 (750–4510)	1780 (1300–2050)	1850 (950–2320)	0.0003
FEV1%	88 (37–130)	90 (45–118)	83 (30–113)	81 (71–93)	47 (37–90)	0.0273
Tiff%	70 (42–86)	76 (51–95)	75 (43–96)	76 (71–81)	56 (38–75)	0.0262
BAL data, median (range)						
Cells/ql	140 (40–520)	110 (40–420)	90 (20–1240)	100 (30–300)	100 (50–320)	0.6347
Viability cells	76.5 (28–92)	78 (50–95)	81.5 (50–96)	84 (50–89)	78 (71–95)	0.7843
Epithelial cells	3 (0–22)	3 (0–21)	2 (1–8)	1.5 (0–3)	3 (0–8)	0.5340
Macrophages	37.5 (14–87)	68.5 (21–94)	85 (8–95)	85.5 (49–91)	61 (47–83)	0.0043
Lymphocytes	38.5 (2–84)	29.5 (5–76)	5.5 (2–29)	7.5 (2–41)	10 (3–14)	<0.0001
Neutrophils	5.5 (1–22)	2 (1–8)	5 (1–11)	6.5 (4–7)	2 (1–3)	0.0002
Eosinophils	1.5 (1–43)	1.5 (1–4)	4.5 (1–78)	2 (1–3)	12 (4–31)	0.0052
CD3	84 (69–95)	91 (74–98)	85 (76–94)	92	Not measured	0.1373
CD4	52 (29–91)	75 (39–89)	50.5 (19–82)	57	Not measured	0.0459
CD8	24 (8–61)	13 (4–38)	35 (12–58)	32	Not measured	0.0115
CD19	0 (0–4)	0 (0–4)	0.5 (0–1)	2	Not measured	0.4972
NK	3 (1–23)	2 (0–5)	2 (1–3)	4	Not measured	0.3755
NKT	3 (2–13)	3 (1–13)	3.5 (2–5)	3	Not measured	0.9581
CD4/8	2.24 (0.48–11.38)	6.45 (1.03–22)	3.58 (0.33–6.83)	1.78	Not measured	0.0099

HP, hypersensitivity pneumonitis; EGPA, eosinophilic granulomatosis with polyangiitis; VC, vital capacity; FEV1, forced expiratory volume in 1 s; BAL, bronchoalveolar lavage; NK, natural killer; NKT, natural killer T. * According to Kruskal–Wallis test (used to compare all 5 groups simultaneously to see whether at least 1 group differs from another) or Chi squared test (sex and smoking; used to compare distributions of categorical variables between all 5 groups).

## Data Availability

The data supporting this study’s findings are available from the corresponding author upon request.

## Supplementary Materials

The following supporting information can be downloaded at: https://www.mdpi.com/article/10.3390/ijms26146831/s1.

## Abbreviations

The following abbreviations are used in this manuscript:

ILD	interstitial lung disease
OLD	obstructive lung disease
HP	hypersensitivity pneumonitis
EGPA	eosinophilic granulomatosis with polyangiitis
BAL	bronchoalveolar lavage
BALF	bronchoalveolar lavage fluid
VC	vital capacity
FEV1	forced expiratory volume in 1 s
NK	natural killer
NKT	natural killer T
IL	interleukin
TNF-α	tumor necrosis factor α
IFN-γ	interferon gamma
VEGF	vascular endothelial growth factor
MCP-1	monocyte chemoattractant protein-1
HRCT	high-resolution computed tomography
TLC	total lung capacity
DLCO	diffusing capacity of the lungs for carbon monoxide
FVC	forced vital capacity
COPD	chronic obstructive pulmonary disease
