# Supplementary material for: Cytokine Profiles of Bronchoalveolar Lavage in Patients with Interstitial Lung Diseases and Non-Allergic Asthma
Source: Int J Mol Sci. 2025 Jul 16;26(14):6831. doi: 10.3390/ijms26146831 (PMC12295261; doi:10.3390/ijms26146831)
Supplement: Supplementary file 1 [file ijms-26-06831-s001.zip › ijms-3707337-supplementary.pdf]

diagnosis	smoking status	CD3	CD4	CD8	CD19	NK	NKT	CD4/8	cells
**HP**	no	86	29	61	4	1	3	0,48	150
**HP**	no	76	49	28	0	1	7	1,75	90
**HP**	no	81	42	41	0	3	5	1,02	220
**HP**	no								120
**HP**		95	91	8	0	1	2	11,38	140
**HP**	no	77	52	19	0	4	4	2,74	120
**HP**	no	91	69	24	0	2	2	2,88	520
**HP**	no	91	74	24	1	5	13	3,08	140
**HP**	no								150
**HP**	no	69	44	22	4	23	3	2	420
**HP**	ex								110
**HP**	no	84	56	25	0	7	3	2,24	40
**Sarcoidosis**	no	below 15%							60
**Sarcoidosis**	no								100
**Sarcoidosis**	no	89	65	23	0	3	5	2,83	50
**Sarcoidosis**	no	92	87	5	1	4	3	17,4	180
**Sarcoidosis**	no	91	61	30	0	2	3	2,03	180
**Sarcoidosis**	no	85	68	15	0	2	4	4,53	50
**Sarcoidosis**	ex	below 15%							70
**Sarcoidosis**	no	85	73	10	0	2	6	7,3	50
**Sarcoidosis**	?	79	39	38	1	3	10	1,03	40
**Sarcoidosis**	ex	below 15%							120
**Sarcoidosis**	no	91	74	18	1	4	3	4,11	270
**Sarcoidosis**	yes								130
**Sarcoidosis**	no	98	79	17	1	3	4	4,65	390
**Sarcoidosis**	no	83	71	11	0	1	5	6,45	120
**Sarcoidosis**	no	96	78	17	1	2	6	4,59	110
**Sarcoidosis**	no	74	48	14	0	1	3	3,43	110
**Sarcoidosis**	no	97	75	19	0	1	12	3,95	70
**Sarcoidosis**	no	93	80	11	0	1	4	7,27	140
**Sarcoidosis**	no								100
**Sarcoidosis**	no								180
**Sarcoidosis**	no	88	76	11	1	2	5	6,7	80
**Sarcoidosis**	no								240
**Sarcoidosis**	no	92	88	4	1	3	1	22	160
**Sarcoidosis**	no	80	57	21	0	2	2	2,71	190
**Sarcoidosis**	?								170
**Sarcoidosis**	yes								60
**Sarcoidosis**	no	97	85	13	0	1	1	6,54	70
**Sarcoidosis**	ex	94	88	5	0	2	1	17,6	60
**Sarcoidosis**	no	95	89	5	0	1	1	17,8	320
**Sarcoidosis**	no	94	84	10	1	3	3	8,4	100
**Sarcoidosis**	no	91	86	7	0	1	3	12,29	130
**Sarcoidosis**	no	94	79	5	0	1	2	15,8	70
**Sarcoidosis**	no								80
**Sarcoidosis**	ex								70
**Sarcoidosis**	yes								210
**Sarcoidosis**	no	92	86	5	1	4	3	17,2	180
**Sarcoidosis**	no	86	75	9	4	1	2	8,33	420
**Sarcoidosis**	yes	89	84	4	0	4	2	21	110
**Sarcoidosis**	no	89	78	10	0	2	3	7,8	80
**Sarcoidosis**	no	84	75	9	3	3	4	8,33	270
**Sarcoidosis**	ex								50
**Sarcoidosis**	no								100
**Sarcoidosis**	no	91	62	22	1	3	13	2,82	170
**Sarcoidosis**	no	87	62	20	0	0	2	3,1	60
**Sarcoidosis**	no	95	70	23	1	2	2	3,04	200
**Sarcoidosis**	no	97	85	11	0	2	2	7,73	130
**Sarcoidosis**	no								100
**Sarcoidosis**	ex								130
**Sarcoidosis**	no								60
**Sarcoidosis**	no	97	81	16	0	1	10	5,06	60
**Sarcoidosis**	no	82	65	13	0	3	5	5	130
**Sarcoidosis**	yes								280
**Sarcoidosis**	no	92	66	25	2	5	6	2,64	170
**Sarcoidosis**	no								70
**Sarcoidosis**	no	92	58	30	1	3	4	1,93	130
**Sarcoidosis**	no	below 15%							90
**Asthma**	no								150
**Asthma**	ex								60
**Asthma**	no								70
**Asthma**	no								90
**Asthma**	no								220
**Asthma**	no								130
**Asthma**	no	94	82	12	0	3	2	6,83	50
**Asthma**	no								340

**Asthma**	ex								1240
**Asthma**	no	76	19	58	1	1	5	0,33	150
**Asthma**	no								40
**Asthma**	no								50
**Asthma**	no								20
**Asthma**	ex								90
**Amiodarone L**	no								300
**Amiodarone L**	no								280
**Amiodarone L**	no	92	57	32	2	4	3	1,78	80
**Amiodarone L**	no								30
**Amiodarone L**	no								100
**EGPA**	no								50
**EGPA**	?								90
**EGPA**	no								180
**EGPA**	no								320
**EGPA**	no								100

viability	ephitelial	macrophages	Lym.	Neutr.	Eos.	IL-8
60	2	14	84	1	1	39,58
74	5	49	29	22		193,41
86	2	20	75	3	2	37,24
92	2	20	14	21	43	66,69
75	4	28	60	8	1	34,81
28	3	33	51	7	9	25,07
92		76	16	4	1	54,86
63	3	42	48	10		46,10
78	1	87	9	2	2	18,88
92	0	19	75	1	1	14,86
86	8	76	2	16	5	35,43
57	22	73	23	2	1	12,06
57	21	62	36	2		46,04
67	1	83	14	3		15,60
78	7	80	19	1		15,89
92	3	66	31	3		11,58
70	2	47	51	1	1	8,04
70	6	74	26			12,44
95	4	21	67	7	1	46,15
75	5	60	37	1	2	14,97
57	6	69	30	1		9,92
75	3	44	56			12,71
86	3	23	76	1		19,18
71	4	94	5	1		16,37
78	1	45	48	5	2	14,14
79	1	83	16	1		25,12
93	1	72	25	1	2	11,28
75	1	67	29	1	3	15,54
67	6	47	50	3		19,83
78	2	71	27	2		31,62
69	5	84	11	1	4	13,22
76	8	86	13	1		24,27
60	4	64	34	2		22,70
79	1	85	15			16,92
92	1	39	59	1	1	11,75
70	2	73	25	1	1	9,42
88	1	89	7	4		17,24
50	4	51	44	5		17,53
69	1	73	25	2		9,03
67	8	63	33	4		27,60
69	1	24	76			15,48
83	3	66	30	3	1	14,70
69	5	72	26	1	1	9,31
67	5	55	44	1		17,73
82	3	91	8	1		21,48
80	8	86	13	1		14,15
82	1	90	8	2		115,45
54	3	63	35	2		16,45
87	2	36	60	1	3	19,26
50	3	26	73		1	13,29
80	6	68	32			16,61
78	3	46	54			19,02
75	2	86	11	3		25,36
93	6	93	6	1		14,09
89	3	61	34	3	1	37,87
86	5	50	49	1		28,88
78	2	78	19	2	1	11,31
82	3	35	63	1	1	13,41
89	2	86	14			20,50
80	3	86	12	2		11,04
86	6	90	8	2		13,39
50	5	48	52			17,67
87	2	58	39	1	2	28,80
94	1	89	9	2		25,18
88	4	60	36	4		8,37
75	0	83	12	2	3	25,50
88	3	72	18	8	2	20,49
89	3	79	15	1	4	24,87
82	2	95	2	2	1	14,10
75	3	77	9	11	3	48,11
70	2	90	5	5		61,22
78	8	92	6	2		21,71
96	1	95	3	1		17,14
90	5	91	4	4		22,82
92	5	78	17	5		16,27
94	1	80	14	6		27,77

81		8	5	9	78	9,16
84	8	53	29	11	7	198,82
75						120,37
83	1	80	6	11	3	16,15
50						76,68
74	2	90	2	1	6	62,25
89	3	90	6	4		60,00
84	1	91	2	6	1	35,35
88	2	49	41	7	2	15,93
50						82,88
80	0	81	9	7	3	31,98
71	3	83	10	3	4	13,60
83	2	47	14	32	7	47,46
78	6	61	5	22	12	70,17
95	0	50	12	6	31	161,93
74	8	73	3	1	23	36,17

IL-1β	IL-6	IL-10	TNF	IL-12p70	IL-2	IL-4
17,93	11,05	8,20	8,78	8,55	10,89	9,43
49,84	10,69	8,26	7,47	8,60	7,95	7,01
10,26	9,84	7,99	8,15	9,58	6,32	6,34
11,68	11,49	7,56	7,61	8,23	10,30	10,23
10,20	10,11	7,80	7,81	8,27	8,81	7,93
30,21	8,73	6,96	6,61	8,03	8,41	7,14
10,35	14,62	7,57	5,96	7,50	7,67	8,04
13,64	19,74	9,72	9,00	10,84	8,95	9,75
10,91	10,44	9,52	9,18	9,55	7,93	7,46
10,58	12,31	9,22	8,18	11,47	7,95	6,91
11,28	11,55	8,94	9,30	9,90	7,44	6,85
8,30	7,45	6,65	6,42	7,58	9,96	9,12
8,71	7,78	6,27	6,51	7,68	8,21	8,21
11,45	9,60	7,91	7,70	9,50	8,56	8,37
10,10	9,93	8,45	7,84	9,33	9,37	8,98
9,78	11,49	7,58	8,19	8,08	8,72	8,2
8,45	7,68	7,12	6,78	7,34	8,55	7,03
11,45	8,68	9,83	7,67	9,13	8,29	7,77
11,70	14,40	9,44	9,60	11,02	9,31	12,41
17,65	11,15	10,43	9,72	8,37	8,92	8,89
8,15	7,65	5,73	6,01	7,23	8,02	6,89
9,58	8,98	6,53	6,78	8,37	7,90	8,77
9,33	10,26	6,49	6,81	7,60	8,40	6,74
9,72	9,26	7,77	7,06	8,61	8,23	9,49
9,69	10,27	7,84	8,25	10,02	8,73	9,03
9,28	8,97	6,20	6,39	7,70	8,36	7,77
9,40	9,48	7,88	8,20	8,75	9,23	9,07
8,22	7,93	6,51	6,35	8,23	8,52	7,36
10,11	9,84	7,99	7,27	9,30	9,67	8,79
9,32	8,77	6,09	6,72	7,08	8,70	8,01
10,54	9,77	7,75	7,84	8,98	8,19	9,69
10,74	8,97	6,03	6,79	8,20	7,89	8,01
11,56	9,81	6,91	7,04	7,96	9,38	7,48
10,92	9,07	7,80	7,44	8,40	7,75	8,11
10,68	10,12	8,18	8,61	10,14	8,68	7,98
9,28	8,01	6,79	6,27	7,46	7,58	7,7
12,34	10,67	9,17	8,29	9,49	8,72	8,32
10,13	9,45	8,92	8,62	9,46	9,30	8,32
8,63	7,76	6,66	6,45	7,11	9,05	8,59
11,18	10,35	8,07	7,53	9,45	8,13	9,45
9,32	8,80	6,86	7,15	7,01	8,98	11,59
10,46	9,85	8,30	6,85	8,71	9,29	8,57
7,73	7,29	5,54	5,59	6,36	9,70	8,77
10,53	11,85	9,00	8,07	8,79	10,64	6,97
9,31	9,23	7,52	7,73	8,31	8,07	9,07
8,89	7,62	6,85	6,45	7,47	8,60	8,29
34,66	11,03	9,22	7,71	8,70	7,74	8,4
11,78	9,49	8,94	8,71	8,76	9,28	9,9
10,03	12,23	7,69	7,11	8,14	9,39	11,33
8,96	8,98	6,65	6,68	7,22	8,22	7,86
11,27	9,94	8,21	8,25	8,30	8,22	8,83
9,76	8,25	7,25	6,28	7,56	7,42	7,65
9,48	8,78	7,90	7,45	8,56	10,96	7,02
9,28	8,25	7,32	6,75	7,98	7,98	8,42
17,20	12,39	12,20	10,58	14,09	7,76	7,69
12,75	11,98	10,67	9,50	11,28	6,61	6,83
10,52	9,15	8,63	7,32	9,25	7,34	7,34
11,22	12,48	9,37	8,35	10,08	10,87	9,12
12,10	9,66	8,19	8,27	8,72		
9,74	8,11	7,16	6,92	7,74	8,50	6,95
11,82	8,75	9,19	7,86	10,51	8,98	8,14
12,12	10,28	9,48	10,06	11,11	12,49	8,32
12,72	11,53	9,43	7,94	9,45	9,78	7,33
13,05	11,39	10,01	7,67	11,27	8,51	8,12
10,20	9,14	8,52	7,88	9,19	9,01	9,56
11,36	10,63	8,36	7,96	9,43	8,56	8
10,24	9,11	8,23	8,60	8,84	7,45	6,64
10,67	11,20	8,15	8,50	8,97	5,81	5,47
12,01	11,27	9,22	8,73	9,75	6,81	6,82
9,95	9,92	8,45	8,10	9,46	6,72	6,29
12,16	17,59	7,80	8,22	9,85	6,61	6,22
10,03	8,39	7,88	7,63	9,94	7,07	6,44
11,40	9,69	8,32	8,53	8,75	8,92	9,32
10,55	11,39	7,21	8,39	8,98	7,19	5,75
10,22	9,88	7,73	8,79	9,29	8,48	8,53
10,12	9,40	7,72	8,31	9,15	9,29	10,22

8,94	29,18	7,27	8,51	7,95	6,72	6,62
21,92	15,05	13,80	10,67	14,80	10,85	8,06
10,56	9,79	7,64	7,78	9,31	7,82	7,24
10,62	9,86	8,51	8,12	10,45	7,69	7,03
11,60	9,79	7,31	7,98	8,79	8,61	7,51
13,54	15,57	10,73	8,81	11,35	10,14	8,66
10,82	9,93	7,64	7,61	9,23	7,73	6,6
11,61	9,80	8,29	8,07	8,17	7,55	7,21
11,45	17,66	8,66	8,52	9,86	8,08	7,31
12,24	9,66	8,20	8,42	9,67	7,07	6,74
12,43	10,80	9,01	9,39	10,12	8	8,17
10,23	9,08	8,28	8,05	8,95	6,88	6,43
9,55	9,00	8,04	8,13	8,95	6,33	6,06
11,59	14,70	8,15	7,72	8,46	46,9	6,56
11,32	11,20	7,27	6,53	8,27	7,92	8,09
10,09	12,20	7,76	7,68	9,28	9,58	9,49

VEGF	ANGIO	RANTES	IFN-γ	C4	C3	C5
15,49	773	653,59	13,02	323,45	78,98	830,3
22,79	514,25	57,76	10,2	298,94	74,56	710,95
13,2	278,39	397,19	9,3	306,56	63,78	781,03
15,76	861,53	55,92	12,89	376,59	99,7	574,72
14,98	564,69	61,89	10,29	469,81	67,01	1791,94
14,45	860,73	172,31	9,89	272,87	47	1692,09
16,98	1384,55	228,41	9,26	239,28	35,93	903,95
16,46	485,71	99,54	11,58	228,59	41,84	552,99
16,07	409,46	33,11	10,2	94,97	35,21	446,46
18,53	1402,29	50,37	9,96	677,96	65,9	2433,55
12,73	296,89	12,26	9,08	144,6	58,18	217,66
14,26	266,87	55,81	10,29	267,53	61,1	1357,17
15,67	1072,32	57,79	11,83	312,4	68,58	722,23
22,73	269,26	19,4	11,42	82,73	61,23	256,81
25,72	287,82	28,63	11,13	561,4	100,65	447,32
24,5	163,42	17,11	11,49	267,19	67,05	1138,45
16,78	125,11	48,44	10	136,43	80,57	300,6
22,82	281,93	21,57	8,9	89,85	66,08	257,66
15,35	556,15	81,42	11,36	271,6	47,46	1582,53
19,09	824,95	36,04	10,93	299,95	86,91	1060,85
22,51	161,43	25,3	10	184,24	56,46	569,77
17,43	261,58	41,29	9,56	264,78	55	3483,27
19,62	348,96	36,53	10,1	265,84	42,83	1496,11
14,62	208,02	17,63	10,62	73,27	58,68	60,63
21,54	401,47	39,42	10,69	248,04	53,88	3564,45
23,18	480,02	30,86	10,47	283,06	68,56	1230,63
33,46	266,44	17,44	10,91	289,77	50,05	1058,62
29,04	651,38	65,31	10,86	291,58	57,55	2240,64
20,95	574,52	41,95	11,99	211,52	47,5	1126,06
35,27	413,26	28,71	10,7	493,07	73,27	2280,11
15,57	1585,54	147,3	10,71	216,52	52,25	1037,75
17,13	247,6	28,73	10,14	226,27	57,9	462,72
22,97	463,55	28,42	10,18	199,78	60,98	400,77
20,61	233,8	41,88	10,44	73,24	55,73	185,49
21,81	212,02	13,9	9,9	269,45	49,81	3041,04
16,72	319,46	54,25	9,4	182,13	52,39	919,51
20,04	340,59	13,62	10,71	93,01	52,74	98,89
18,26	140,95	12,28	11,42	135,71	55,83	318,15
16,09	93,63	12,83	12,21	150,67	54,72	117,82
17,97	320,97	22,22	10,88	194,78	64,59	274,72
24,51	651,25	63,08	10,56	216,41	38,89	1374,07
22,12	264,69	38,18	12,14	156	70,72	524,77
25,01	214,17	17,81	10,76	216,39	51,32	931,98
21,23	263,42	58,68	11,81	236,42	51,77	932,16
20,94	362,31	39,2	9,93	155,66	57,43	342,25
19,59	260,9	27,57	11,72	100,47	55,3	242,67
17,77	932,74	40,55	10,13	150	47,22	231,26
20,68	602,38	135,04	9,83	291,62	43,3	898,46
16,12	636,88	61,71	11,37	390,21	44,94	1897,6
15,62	368,53	66,47	10,43	199,44	41,49	1217,21
24,36	423,6	32,02	9,53	193,36	44,19	905,09
21,9	418,9	37,87	9,49	243,23	46,16	1163,91
21,08	328,05	17,68	9,37	169,71	46,67	351,34
26,94	197,97	25,68	10,56	144,72	46,23	270,82
31,16	646,37	24,59	9,8	215,94	54,07	1138,49
14,04	279,84	30,59	9,39	187,61	49,95	564,33
24,31	181,42	14,05	9,61	156,55	52,5	507,39
22,73	354,36	18,84	9,47	216,38	48,84	3539,38
22,29	164,22	21,27	9,92	149,34	59,99	463,04
28,23	218,4	12,65	9,93	117,57	57,9	229,16
22,75	190,86	24,03	12,08	128,92	52,29	145,65
22,86	275,75	20,49	11	187,14	59,41	488,35
21,41	1011,18	20,39	10,23	160,26	51,16	492,25
31,98	349,01	26,39	11,62	194,99	46,3	453,72
19,33	667,74	20,34	11,21	130,61	54,54	187,96
19,69	241,49	15,17	8,41	189,94	61,71	216,6
22,64	450,7	11,6	7,82	141,74	59,48	278,23
13,72	131,73	13,5	8,66	152,82	89,17	64,72
20,62	467,42	130,32	8,74	316,86	94,54	1291,93
24,42	1510,49	228,25	8,58	380,03	42,08	1710,99
32,22	291,76	31,58	9,1	115,04	86,43	298,53
13,65	221,28	16,2	10,62	124,2	58,54	80,6
13,75	230,35	26,82	9,45	165,4	56,94	196,78
28,8	277,23	26,72	10,95	118,45	63,7	236,07
15,8	241,54	23,59	11,25	208,98	49,55	963,92

12,82	1087,46	54,94	9,05	373,91	66,15	1858,29
22,95	1164,95	92,34	10,42	211,51	66,54	539,58
17,46	1050,7	97,47	10,06	171,43	75,8	158,2
18,87	508,12	14,14	8,93	223,2	67,98	456,62
15,81	322,38	15,08	10,25	175,67	98,88	155,53
17,09	2160,84	136,93	11,94	139,64	52,63	352,4
13,03	516,23	39,32	8,34	247,06	110,85	302,59
15,81	764,42	80,23	9,78	205,84	55,69	689,14
13,98	646,54	107,33	9,63	213,9	50,17	978,8
15,55	1886,4	76,32	8,26	182,35	93,61	642,38
14,88	397,6	85,61	11,21	324,69	55,05	995,19
16,73	596,18	37,02	8,83	148,08	72,86	243,93
13,3	1171,64	250,98	8,1	379,27	57,74	2949,3
18,18	1974,89	90,05	8,57	509,38	73,52	2820,04
20,53	547,87	40,9	9,75	305,16	74,58	1252,59
17,41	1254,58	87,54	11,2	288,1	85,95	846,71
